# A novel T4- and λ-based receptor binding protein family for bacteriophage therapy host range engineering

**DOI:** 10.3389/fmicb.2022.1010330

**Published:** 2022-10-31

**Authors:** Samuel J. Magaziner, George P. C. Salmond

**Affiliations:** Department of Biochemistry, University of Cambridge, Cambridge, United Kingdom

**Keywords:** bacteriophage, receptor binding proteins, antimicrobial resistance, phage therapy and biotechnology, phage T4, phage λ, *Citrobacter rodentium*

## Abstract

Widespread multidrug antimicrobial resistance in emerging pathogens has led to a renewed interest in phage therapy as an alternative or supplement to traditional small molecule drugs. The primary limiting factors of phage therapy deployment rest in the narrow host range specificity of phage as well as a poor understanding of many phages’ unintended downstream effects on host physiology and microbiota as well as on adverse pathogen evolution. Consequently, this has made assembling well-defined and safe “phage-cocktails” of solely naturally occurring phages labor- and time-intensive. To increase the speed, efficacy, and safety of therapeutic deployment, there is exceptional interest in modulating the host ranges of well-characterized lytic phages (e.g., T4 and T7) by using synthetic strategies to the swap phage tail components, the receptor binding proteins (RBPs) key for host specificity. Here we identify the RBP of the *Citrobacter rodentium* temperate phage ΦNP as ORF6. Through bioinformatic and phylogenetic assays, we demonstrate this RBP to be closely related to the known RBPs of T4 and λ. Further investigation reveals a novel, greater than 200 members RBP family with phages targeting several notable human pathogens, including *Klebsiella pneumoniae*, *Escherichia coli* O157:H7, *Salmonella* spp., and *Shigella* spp. With well characterized lytic members, this RBP family represents an ideal candidate for use in synthetic strategies for expanding therapeutic phage host ranges.

## Introduction

Bacteriophages, or phages, are obligate intracellular parasites of bacteria. With an estimated global 10^31^ phage particles they are thought to be the most abundant biological entities on Earth and important drivers of bacterial host evolution and population dynamics (Salmond and Fineran, 2015). With the arrival of widespread antibiotic resistance in emerging pathogens (Fischbach and Walsh, 2009; Crofts et al., 2017), specific bacterial targeting and killing with phage therapy, for a means of alternate or joint treatment with wide-spectrum antibiotics, has seen renewed interest with some notable triumphs (Gordillo Altamirano and Barr, 2019; Hatfull et al., 2022; Nick et al., 2022). However, some of the primary challenges facing the effective employment of traditional “cocktail” bacteriophage therapy within a clinical setting are the often highly specific host-range exhibited by a given phage and unintended downstream effects on host physiology and microbiota as well as unintended and adverse pathogen evolution (Sulakvelidze et al., 2001; Lu and Koeris, 2011; Nilsson, 2014). Host-range, defined at the strain level; downstream effects, such as endotoxin release, local inflammatory effect, or off-target microbiota lysis; and temperate or lytic nature can differ from phage to phage, even those targeting the same pathogen. Thus, there exist impracticalities behind the assessment of every newly isolated phage with enough detailed examination for safe, efficient, and speedy deployment in a human system.

Recently, advances in genome engineering and synthetic biology methodologies have provided an alternative to traditional bacteriophage therapy. Instead of employing a “cocktail” of naturally occurring phages of different genomic compositions and with unknown downstream effects, researchers have pursued phage genome engineering techniques (Kiro et al., 2014; Martel and Moineau, 2014) to generate single, well-characterized phages capable of targeting multiple, specific bacterial hosts through exploitation of receptor binding protein (RBP) modularity. Bacteriophage host recognition and binding (adsorption) to a cognate bacterial receptor is the first stage of phage infection and a primary limiting factor for phage propagation and host range determination. Receptors exploited by phages can be any component of the bacterial surface including capsular polysaccharides, residues of the lipopolysaccharide (LPS) in Gram-negatives, flagella, pili, or surface membrane proteins (Silva et al., 2016; Tzipilevich et al., 2017). Some phages employ a dual-receptor model of adsorption, such as phage T4 which requires both specific LPS residues as well as the outer membrane protein, OmpC, to infect (Henning and Jann, 1979; Washizaki et al., 2016). Phages recognize and bind to their host receptors through RBPs. Depending on phage morphology these are most often named tail-spikes, spike proteins, or tail fibers (Silva et al., 2016). The role of RBPs is to facilitate the adsorption and proper orientation of phages on the extracellular surface to initiate infection, with RBP–receptor interactions often resulting in a cascade of structural rearrangements, from baseplate alteration to tail sheath contraction (Hu et al., 2015). Phage RBPs have been shown to be very stable proteins and are highly resistant to proteases and detergents, presumably required for survival and functionality in harsh environments, such as the mammalian intestine. Moreover, RBPs can exhibit tight receptor specificity and affinities comparable to those of antibodies or lectins, making them particularly interesting molecules (Simpson et al., 2016).

Earlier studies focusing on the T3 and T7 phage families, successfully conferred expanded phage host ranges to lytic phage virions by the swapping of RBP tail fiber regions for phages targeting pathogenic *Klebsiella* spp. and *Yersinia* spp. (Ando et al., 2015; Yosef et al., 2017). Other strategies, such as those explored by Ram, Ross, Novick and colleagues, focused on developing “antibacterial drones,” engineered and non-replicative phage particles lacking the ability to package endogenous phage DNA, to deliver antibacterial staphylococcal pathogenicity island cargo genes to a cognate bacterial target; this approach both cleared *Staphylococcus aureus* in mice infection while simultaneously mitigating unintended downstream effects of more traditional biologically-active phage therapies (Ram et al., 2018). While successful, expansion of these approaches to other pathogens or even strains of the same species would likely require a “ground-up” rebuild due to limited tools for enabling phage host-range versatility. Indeed, for engineered phage-based therapies to respond effectively to the constantly evolving pathogen landscape, these methodologies will have to be expanded to cover more pathogenic strains and, consequently, will require finer tuned phage RBP families for host range modulation. Here we identify such an RBP family based in the well characterized T4, Tula, Tulb, λ, and, here identified, ΦNP lineage.

Traditionally, *de novo* identification of phage RBPs through known-RBP amino acid or DNA sequence homology analysis has proven difficult. This is in part due to the highly specific nature of each RBP-receptor pairing, phage modularity, the rapid evolution of phage genomes mediated by hyper-modification of phage DNA bases, and the sheer impracticality of examining every new phage at the structural level (Warren, 2003; Lima-Mendez et al., 2011). However, previously, we provided an in-depth characterization of the prophages of the non-motile, Gram-negative, enteric bacterium *Citrobacter rodentium* (Petty et al., 2011; Magaziner et al., 2019). This bacterium is a natural host-adapted intestinal mouse pathogen, causative agent of transmissible murine colonic hyperplasia, and important model organism for the study of enteric pathogens of the attaching and effacing (A/E) family (Barthold et al., 1978; Schauer and Falkow, 1993; Mundy et al., 2004; Wong et al., 2011; Collins et al., 2014). Previously, we showed that the ten prophages (complete and incomplete) of this bacterium represent a conserved family of horizontally-acquired mobile genetic elements associated with enteric evolution towards pathogenicity, playing a key role in horizontal gene transfer (HGT) within the enteric environment and carrying several virulence-associated cargo genes. We also showed that of these ten prophages, two, named ΦNP and ΦSM, actively excise spontaneously to form functional temperate phage particles. Furthermore, we showed that ΦNP and ΦSM utilize residue-specific lipopolysaccharide (LPS) components for adsorption and infection with ΦNP utilizing the Glucose-II (Glc-II, catalyzed by WaaO) residue and ΦSM utilizing the Glucose-III (Glc-III, catalyzed by WaaR) residue of the outermost LPS core sugars (Magaziner et al., 2019).

Here, we employ these prior insights and a suite of bioinformatics assays to identify a structural protein (ORF6) of ΦNP showing extensive protein-level homology to the known RBPs of T4 and λ. Through adsorption kinetics assays and use of a sfGFP-tagged version of this structural protein and predicted tail fiber, we show that ORF6 is the putative RBP of ΦNP. We also show that the known ΦNP, T4, TuIa, TuIb, and Ur-λ RBPs all belong to a conserved family of tail fibers represented in the genomes of phages of several notable human pathogens including *Klebsiella pneumoniae*, *Escherichia coli* O157:H7, *Yersinia pestis*, *Salmonella* spp., and *Shigella* spp. While earlier literature focusing on the structure of the RBP of phage T4 has suggested the possible existence of such a family (Bartual et al., 2010), identification and verification of the RBP of ΦNP as one its members suggests that this greater, here characterized, family of RBPs represents an ideal candidate for use in the design and engineering of therapeutic bacteriophage technologies with expanded host ranges.

## Materials and methods

### Bacterial strains and culture conditions

The bacterial strains used in this study are listed in Table 1. Strains of *Citrobacter rodentium* and *Escherichia coli* were grown at 37°C. Overnight cultures were grown in 5 ml Luria broth (LB) in sterile 25 ml culture tubes placed on a rotary wheel. Bacterial growth was determined by measuring the optical density of the culture at a wavelength of 600 nm (OD_600_) using a Unicam Heλios spectrophotometer and cuvettes with a 1 cm path length. Solid medium contained 1.5% weight by volume (*w*/*v*) agar with soft medium overlay (top agar) using 0.35% agar; both were made with LB unless otherwise noted. For long term storage, 800 μl of overnight cultures were mixed with 200 μl 80% (*w*/*v*) vacuum-sterilized glycerol. Samples were briefly vortexed, appropriately labelled, and stored at −80°C. Phage buffer was composed of 10 mM Tris/HCl pH 7.4, 10 mM MgSO4, and 0.01% gelatin. Phage used in this study are listed in Table 2.

**Table 1:** Bacterial strains used in this study.

Strain	Relevant Characteristics	Source/Reference
***Citrobacter rodentium***		
ICC168	Reference strain	Petty et al. (2011)
***Escherichia coli***		
MG1655	K-12 derivative; F−, λ−*, rph-1*	Blattner et al. (1997)
ER2507	K-12 derivative, F−,*ara-14 leuB6 fhuA2 Δ(argF-lac)U169 lacY1 glnV44 galK2 rpsL20 xyl-5 mtl-5 Δ(malB) zjc::Tn5(KanR) Δ(mcrC-mrr)HB101*	New England Biotech
DH5α	K-12 derivative, F−, *Φ80lacZΔM15, Δ(lacZYA-argF)U169, endA1, recA1, hsdR17 (rk−mk+), deoR, thi-1, supE44, λ−, gyrA96, relA1*	Invitrogen
ER2507(ΦNP)	ER2507 ΦNP lysogen	Magaziner et al. (2019)
ER2507(ΦSM)	ER2507 ΦSM lysogen	Magaziner et al. (2019)
BW25113	K-12 derivative, *rrnB3, ∆lacZ4787, hsdR514, ∆(araBAD)567, ∆(rhaBAD)568, rph-1*	Baba et al. (2006)
JW3602-1	BW25113 ∆*waaO*::*kan*	Baba et al. (2006)

**Table 2:** Bacteriophages used in this study.

Phage	Description	Origin	Reference
ΦNP	Temperate phage of *C. rodentium*	*C. rodentium* supernatant	Petty et al. (2011)

### Generation of high titer phage stock

A high titer phage sample was obtained by collecting top lawns displaying near confluent lysis into a glass universal container. The plate surface was then washed with 3 ml phage buffer and mixed with harvested top agar in the glass universal. Next, 500 μl of chloroform was added and the sample vortexed vigorously for 5 min. Following a sitting incubation period of 15–20 min at room temperature, the sample was spun down at 2220 × *g* and 4°C for 20 min. The resultant supernatant was then removed, transferred to a new glass bijou container, mixed with a drop of chloroform to ensure sample sterility, and stored at 4°C until use. These steps were then repeated until a viral stock yielded a titer of > 10^9^ pfu/ml and then purified.

### Phage purification

This protocol was undertaken as previously described (Boulanger, 2009). In brief, PEG-8000 was used to precipitate phage particles overnight from a high titer lysate. 5–6 confluent top lawns containing the phage of interest were harvested, extracted with chloroform and incubated with DNase I (1 μg/ml) and RNase A (1 μg/ml) for 30 min at room temperature. NaCl was dissolved into the lysate to the concentration of 0.5 M and left to cool for 1 h at 4°C. Following centrifugation at 2220 × *g* for 10 min and 4°C, the crude lysate was filter sterilized and PEG-8000 was added to a final concentration of 10% and mixed until dissolved. This mixture was kept overnight at 4°C. The precipitated phage was then gently pelleted, supernatant removed, pellet resuspended in phage buffer (10% of original volume), and extracted with chloroform to yield PEG purified phage lysate. PEG precipitated phage lysate was further purified *via* a step gradient CsCl centrifugation followed by an CsCl isopycnic equilibrium centrifugation step (gradient layers of 1.20, 1.30, 1.40, 1.50, and 1.70 g/ml; isopycnic gradient of 1.50 g/ml). Opalescent bands of phage were aspirated *via* syringe. CsCl was removed from the purified phage solution *via* dialysis with a 10 kDa MW cutoff membrane overnight at 4°C in 10,000x volume phage buffer All ultracentrifugation steps were performed at 35,000 rpm (~150,000 × g) carried out in a Beckman Coulter Optima L-100 XP ultracentrifuge with a SW-40ti swinging bucket rotor.

### Phage genomic DNA extraction

Phage DNA was extracted using a phenol-chloroform protocol. In a phase-lock gel (PLG) tube, 450 μl of high titer phage lysate was incubated with 4.5 μl of 1 mg/ml DNase I and 2.5 μl of 10 mg/ml RNase A and incubated at 37°C for 30 min. The mixture was then added to 11.5 μl of 20% SDS and 4.5 μl of 10 mg/ml Proteinase K and incubated for another 30 min. DNA was extracted by adding 500 μl of a phenol:chloroform:isoamyl alcohol 25:24:1 mix and centrifuged at 1500 × *g* for 5 min. The supernatant was transferred to a new PLG tube and the previous step repeated. In a new PLG tube, the supernatant was supplemented with 500 μl of chloroform:isoamyl alcohol 24:1 and centrifuged at 1,500 × *g* for 5 min. The aqueous phase at the top was then incubated with 45 μl sodium acetate (3 mol/l, pH 5.2) and 500 μl of 100% isopropanol at room temperature for 45 min. The mixture was then subjected to centrifugation at 12,000 × *g* for 20 min, after which the pellet was washed at least twice with 70% ethanol and then re-suspended in dH_2_O.

### Phage chemical mutagenesis

Chemical mutagenesis was conducted utilizing hydroxylamine containing phosphate-EDTA buffer as previously described (Villafane, 2009).

### DNA manipulations, oligonucleotides, and sequencing

Unless otherwise stated, standard molecular biological methods were used for all DNA manipulations. Genomic and plasmid DNA were purified using the GeneJET Genomic DNA Purification Kit (Thermo Scientific) and GeneJET Plasmid Miniprep kit (Thermo Scientific) according to manufacturers’ instructions. All restriction enzymes used were obtained from New England Biolabs and used according to manufacturer’s protocols. DNA fragments were ligated using T4 DNA ligase (NEB). Oligonucleotides were obtained from Sigma Aldrich and are listed in Table 3. DNA sequencing of PCR and plasmid products was performed by GATC Biotech utilizing their Lightrun Tube-Barcode Sanger Sequencing.

**Table 3:** Oligonucleotides used in this study.

Name	Sequence (5′-3′)	Comments	References
PF106	GACCACACGTCGACTAGTGCNNNNNNNNNNAGAG	RP-PCR primer 1	Fineran et al. (2005)
PF107	GACCACACGTCGACTAGTGCNNNNNNNNNNACGCC	RP-PCR primer 2	Fineran et al. (2005)
PF108	GACCACACGTCGACTAGTGCNNNNNNNNNNGATAC	RP-PCR primer 3	Fineran et al. (2005)
PF109	GACCACACGTCGACTAGTGC	RP-PCR adapter primer	Fineran et al. (2005)
oREM7	CTAGAGTCGACCTGCAGGC	pDS1028 replicon clone sequencing primer	Monson et al. (2015)
SM.P45	TGCAATCTAAAACTAGTAACATGCGTAAAGGCGAAG	f; sfGFP addition of *Spe*I cutsite	
SM.P46	CAATTTTTTGGAATTCACCAGAACCCGCCGCAGAACCCG CAGAACCTTTGTACAGTTCATCCATACC	r; sfGFP addition of Linker and *EcoR*I cutsite	
SM.P47	GCGGGTTCTGGTGAATTCCAAAAAATTGGAGATCTCA	f; ΦNP ORF 6 addition of Linker and *EcoR*I cutsite	
SM.P49	TCGATATCAAGCTTTTATGCAAGCCTCACGAT	r; ΦNP ORF 6 addition of *Hin*dIII custsite	
SM.P50	GCTTGCATAAAAGCTTGAAGGCATCAAATAAAACGAAAG	f; pZA11 addition of *Hin*dIII cutsite	
SM.P51	TAAGTCTAGTTACTAGTTGATTTTCTCCTCTTTGTGC	r; pZA11 addition *Spe*I cutsite	

### Generation of plasmids used in this study

The plasmids used in this study are listed in Table 4. pZSM1 was generated as a derivative of the pZA11 background, adding, in front of the native pL-TetO Tc repressible promoter and RBS a SpeI cutsite and a terminal *Hin*dIII cutsite using primer SM.P51 and SM.P50, respectively. Next, the sfGFP element was extracted from pZA11 with primers SM.P45 and SM.P46 adding a 5′ *Spe*I cutsite and a 3′ flexible amino acid linker sequence (N′-GSAGSAAGSGEF-C′) with a 3′ *Eco*RI and *Hin*dIII cutsite. These two elements were then digested, ligated, and cloned to form vector pZSM1. Next, ΦNP ORF6 was extracted from phage gDNA with primers SM.P47 and SM.P49 adding a 5′ *Eco*RI cutsite and a 3′ *Hin*dIII cutsite, respectively. Lastly, ORF6 and pZSM1 were digested using *Eco*RI and *Hin*dIII, ligated, and cloned to yield vector pZSM6b. Both vectors were sequence verified and assessed under light microscopy to verify the intact nature of the sfGFP marker.

**Table 4:** Plasmids used in this study.

Plasmid	Relevant Characteristics	References
pZA11	High copy Ap^r^, sfGFP expressing plasmid	The Wang Lab, Columbia University
pBAD33	Arabinose-inducible Cm^r^ plasmid	Guzman et al. (1995)
pZSM1 (sfGFP-L)	pZA11 derived plasmid containing a sfGFP and flexible linker sequence (N′-GSAGSAAGSGEF-C′) under a tetracycline repressible promoter pL-TetO; Ap^r^	This study (Chen et al., 2013)
pZSM6b (sfGFP-L-ORF6)	pZSM1 derivative containing ΦNP ORF 6 sfGFP fusion construct	This study

### Fluorescence binding assay of sfGFP-L-ORF6

Overnight cultures (5 ml) containing sfGFP-L-ORF6 and sfGFP-L were either chloroform treated or sonicated for a 1 min at 15 micron amplitude with a Soniprep 150 (MSE). Chloroform treated samples were observed immediately by fluorescent light microscopy. Sonicated lysates were spun down at 2220×*g* and 4°C for 10 min, followed by syringe-driven 0.22 μm filter sterilization prior to use. 100 μl of test and control crude protein extract was added to 900 μl of WT ER2507 cells normalized to an OD_600_ of 1.0 and samples taken every 5 min to be analyzed by fluorescence and light microscopy. Sonicated lysates were also plated on LB-agar plates and allowed to grow up overnight at 37°C to ensure observed fluorescence was a product of tail fiber binding and not exogenous, sfGFP+ bacteria.

### Adsorptions assays

Triplicate overnight cultures of strains to be tested were grown up in 5 ml of LB on a tube roller at 37°C. Pre-made Eppendorf tubes containing 900 μl phage buffer and 30 μl chloroform were labelled and set out for each sample and time point to be collected. To each sample of 5 ml overnight bacterial culture, a combination of either (1) ΦNP + LB, (2) ΦNP + ER2507, (3) ΦNP + ER2507 + sfGFP-L lysate, (4) ΦNP + sfGFP-L lysate, (5) ΦNP + ER2507 + sfGFP-L-ORF6 lysate, or (6) ΦNP + sfGFP-L-ORF6 lysate (MOI of 0.01) and mixed immediately. From these mixtures 100 μl was removed for time point 0 min and added to the pre-made sample tubes and quickly vortexed for 5 s. Sample collection occurred in a similar fashion for the next 55 min every 5–10 min. Vortexed samples were then spun down and supernatant removed. These supernatants were then titrated in serial dilution on bacterial top lawns. The final adsorption curve was plotted by calculating the percentage of free phages in the culture against time.

### Bioinformatic analysis

Coding sequences and ORFs were determined by a combination of prior annotations and the Geneious R7 predictive ORF function on known *C. rodentium* IIC168 genome and prophage sequences (accession number: NC_013716; Kearse et al., 2012). Protein functionality and homology were predicted using BLASTP.^1^ Phage protein products of interest were initially identified using blastp, constraining search results to known viral sequences (taxid: 10239). Genome comparisons were generated by the Artemis Comparison Tool (ACT) (Carver et al., 2005) and EasyFigure (Sullivan et al., 2011).

### Phylogenetic analysis

Protein alignments were generated using ClustalOmega (Sievers et al., 2011). Phylogenetic trees were constructed by the maximum-likelihood method under the LG model (Le and Gascuel, 2008) with PhyML (Guindon and Gascuel, 2003) and verified using Bayesian likelihood using Blosum matrix support (Henikoff et al., 1992) plus Gamma model with MrBayes (Huelsenbeck and Ronquist, 2001). PhyML branch support was tested using 1,000 bootstrap replicates with Bayesian modelling further corroborating PhyML branches.

## Results

### ΦNP ORF6 shares high similarity with the known T4 RBP (gp37)

**Figure 1:**
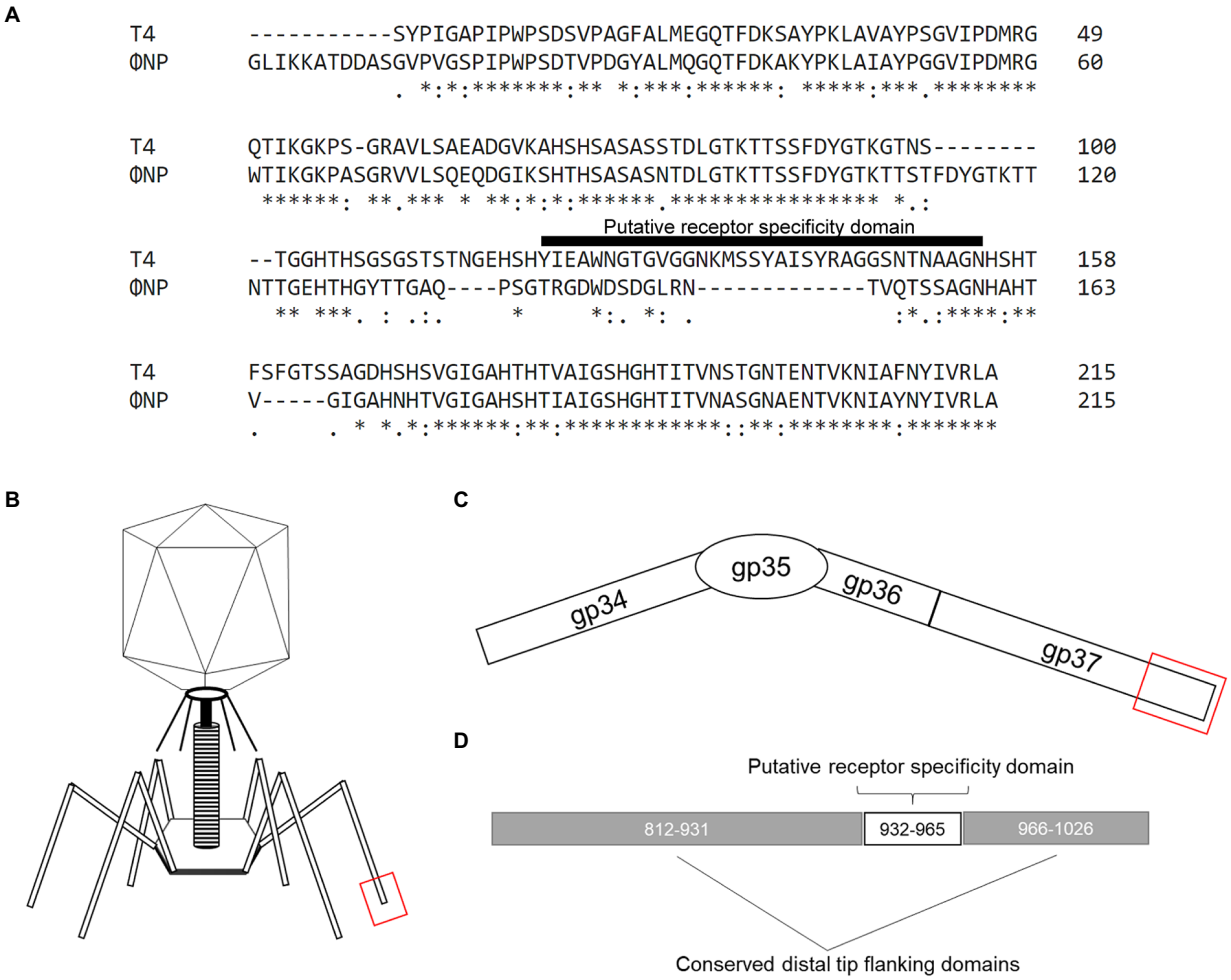
The previously uncharacterized open reading frame 6 (ORF6) of the *Citrobacter rodentium* temperate phage, ΦNP, shares high similarity with the known T4 RBP (gp37). **(A)** Amino acid (AA) alignment by ClustalOmega depicting high similarity between ΦNP ORF6 and the distal 215 AAs of the 1,026 AA-long known T4 RBP (gp37). Note, primary sequence dissimilarity is found at the same site as the putative receptor specificity domain (here noted as T4 AA position 120–154) of gp37. **(B)** A cartoon depicting the structure of a bacteriophage T4 virion. **(C)** A schematic of the bacteriophage T4 tail fiber. The tail fiber is composed of the protein products of gp34, gp35, gp36, and gp37. The RBP element of phage T4 specificity has been isolated to the interaction of gene product gp37’s distal end and bacterial surface protein OmpC. **(D)** A schematic noting the distal 215 AA of gp37 with conserved distal tip flanking domains (AA 812–931 and 966–1,026; as conserved in ORF6 of ΦNP) and putative receptor specificity domain (AA 932–965 of gp37).

To identify the potential RBPs of ΦNP and ΦSM, we utilized the recently reannotated ΦNP and ΦSM genomes (Magaziner et al., 2019) and extracted all coding regions predicted to encode structural proteins. We next took the translated amino acid gene products of these coding regions and searched for notable sequence homology, both within non-specified search parameters as well as within the viral NCBI database (taxid: 10239). Translated coding regions demonstrating protein-level homology to proteins with known or predicted non-RBP functionalities (such as capsid, sheath, or portal proteins) were removed from the analysis. As ΦNP and ΦSM are members of the *Myoviridae* and *Siphoviridae* families, respectively, their RBPs would be expected to be tail fibers or spikes. While no significant tail spike homology was found for the ΦSM structural comparison, one coding region of ΦNP, ORF6 displayed striking homology to a 215 AA stretch of the tail fiber protein product encoded by the phage T4/Tula/Tulb coding regions of gp37 (Figure 1A). ORF6 is encoded antisense in the ΦNP prophage genome from base pair 3,252 to 2,404; this corresponds to base pairs 2,733,682 to 2,732,833 in the *Citrobacter rodentium* ICC168 genome (Genbank accession: FN543502.1).

Notably, the RBP of T4 (and close relatives, phages TuIa and TuIb) is well characterized and has been identified as the gene product of gp37, a 1,026 amino acid (AA) long tail fiber (LTF). Both the structure (Bartual et al., 2010) and putative residues encoding receptor specificity of the T4 RBP (residues 907–996; Montag et al., 1990) have been identified. The general structure of the gp37 LTF is comprised of a six-stranded antiparallel beta-strand needle domain, with three chains intertwining to form a broad head domain, in which the putative receptor binding domain sits (Figures 1B–D; Bartual et al., 2010). Despite gp37 encoding a 1,026 AA LTF, the receptor binding elements comprise only the last 215 residues (Figures 1B–D) and allows specific binding of the T4 bacteriophage to its putative receptor OmpC (with a secondary and reversible affinity for LPS; Washizaki et al., 2016). As previously reported, ΦNP utilizes Glucose-II (Glc-II, catalyzed by WaaO) of the LPS for absorption and infection (Magaziner et al., 2019).

Interestingly, ORF6 was predicted to encode a protein comprised of 282 AAs. Despite this notable difference in tail fiber size, a high degree of similarity could be seen between the gene product of ORF6 and that of gp37. The majority of identical or similar residues were located between the last 215 residues of both gene products representing the distal tail fiber tip with a large region of dissimilarity observed within the putative receptor specific region of the T4 LTF (residues 907–996, noted in Figure 1A as residues 121–154). These data suggested that ORF6 was a good candidate for the RBP of ΦNP.

### ORF6 encodes the RBP of ΦNP

**Figure 2:**
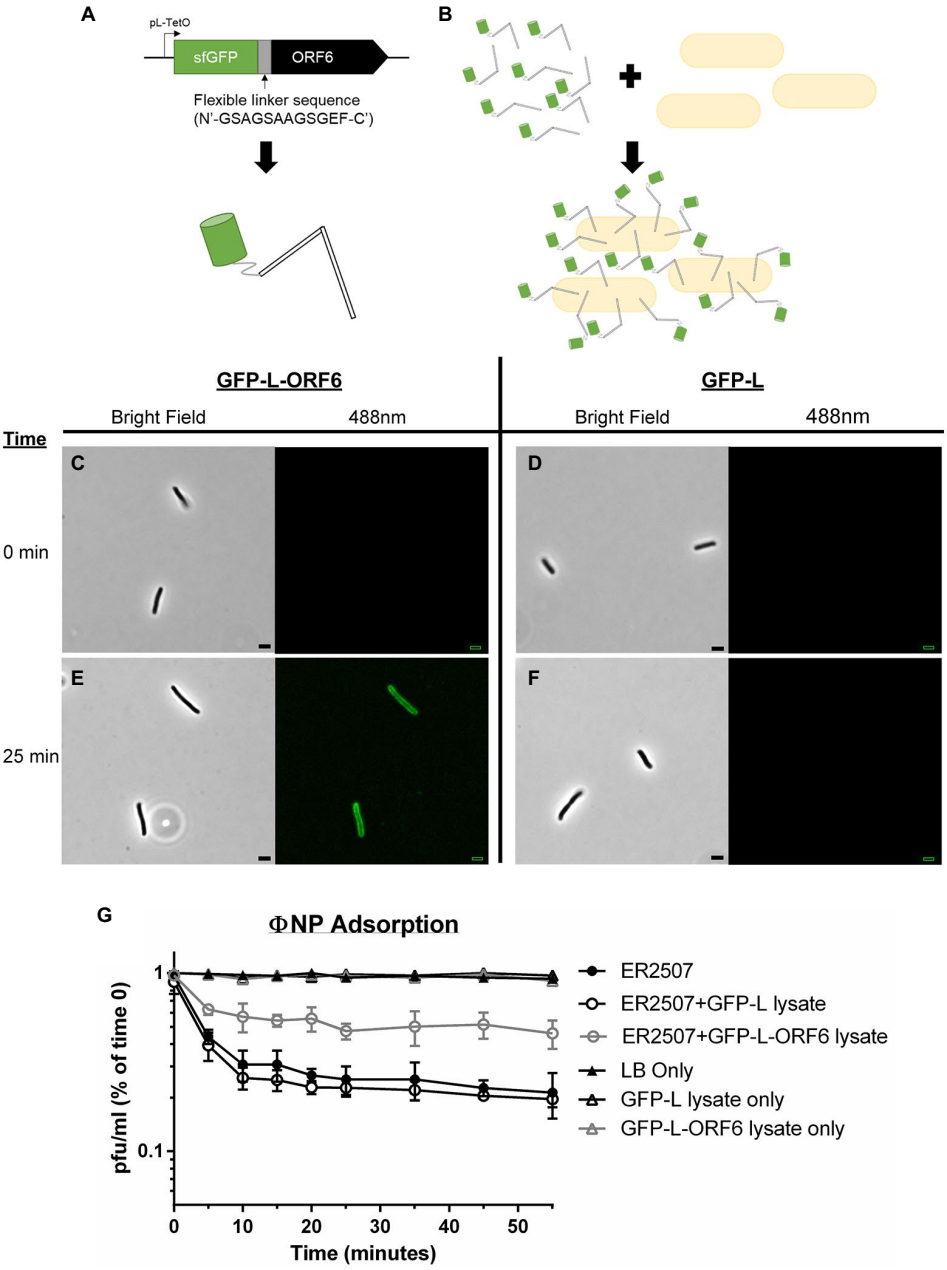
Adsorption kinetics and fluorescence assays show that ORF6 encodes the putative RBP of ΦNP. **(A)** A cartoon depicting the construct utilized to assess ORF 6 binding. ORF6 was recombinantly expressed with a superfolder GFP (sfGFP) fusion tag, linked with a flexible linker sequence (N′-GSAGSAAGSGEF-C′), and under control of a pL-TetO Tc repressible promoter (named sfGFP-L-ORF6). As a control an identical construct lacking ORF6 was built (named GFP-L). **(B)** Cartoon depicting fluorescence localization to the surface of host bacteria (not to scale). **(C-F)** Fluorescence localization assay of crude sfGFP-L-ORF6 lysate **(A,E)** and control crude GFP-L lysate **(D,F)** using host bacteria *E. coli* K12 strain ER2507 under both brightfield and 488 nm emission. Notably, at 0 min **(C,D)** no background fluorescence is seen in either sample. Following 25 min **(E,F)** fluorescence can be seen localized to the periphery of bacteria in the sample containing crude sfGFP-L-ORF6 lysate **(E)** while none is seen with samples containing GFP-L crude lysate **(F)**. **(G)** Representative graph showing the mean results of triplicate adsorption assays of ΦNP over 55 min onto *E. coli K-12* strain ER2507 in the presence of no lysate, crude sfGFP-L-ORF6 lysate, or crude sfGFP-L lysate with error bars denoting SD. Also shown are the results of *E. coli K-12* strain ER2507 in the presence of no lysate, crude sfGFP-L-ORF6 lysate, or crude sfGFP-L lysate with no addition of ΦNP. As can be seen, addition of GFP-L-ORF6 lysate to of *E. coli K-12* strain ER2507 in the presence of ΦNP greatly reduces ΦNP adsorption kinetics. The left axis shows phage left unabsorbed as a percentage of phage at time 0 min in pfu/ml.

To determine whether ORF6 was indeed the RBP of ΦNP, ORF6 was recombinantly expressed with a superfolder GFP (sfGFP) fusion tag, linked with a flexible linker sequence (N′-GSAGSAAGSGEF-C′; sfGFP-L-ORF6; Figure 2A; Chen et al., 2013). As a control, an identical vector and linker sequence, but lacking the ORF6 insertion, was utilized (sfGFP-L).

ΦNP host range has been shown to include *E. coli* K12 strain ER2507 (Magaziner et al., 2019). To examine ORF6 as the potential RBP of ΦNP, the ability of sfGFP-L-ORF6 to localize to the extracellular surface of wild type (WT) ER2507 in culture was examined (Figures 2A–E). A crude protein extract was mixed with an ER2507 WT liquid culture and fluorescence localization was visualized over a time course of 55 min (Figure 2B). While no detectable fluorescence localization was seen between 0 and 20 min (Figure 2C), after 25 min (Figure 2E), most cells within the test culture displayed an apparent fluorescent localization. Notably, fluorescent localization was absent in the sfGFP-L control (Figures 2D,F). Previously we had identified the ΦNP bacterial surface receptor as the lipopolysaccharide (LPS) residue GlcII, a residue dependent on the catalytic activity of the *waaO* gene in the core LPS biosynthesis cascade (Magaziner et al., 2019). As expected, no fluorescent localization was observed in tests conducted utilizing sfGFP-L-ORF6 with the ΦNP receptor knockout strain (Δ*waaO*; data not shown).

Lastly, the effect of sfGFP-L-ORF6 on the adsorption of ΦNP was examined. Were ORF6 the RBP of ΦNP, it would likely compete with active phage virions for extracellular binding sites, lowering overall adsorption kinetics and total phage binding. An adsorption test was performed under standard conditions with or without the addition of a sfGFP-L-ORF6 crude protein extract. In the presence of GFP-L lysate, phage adsorption was consistent with WT (ER2507 only) kinetics and observed to plateau near 25 min (Figure 2G), consistent with observation of fluorescence localization of the sfGFP-L-ORG6 lysate. However, the presence of the sfGFP-L-ORF6 in the adsorption assay was found to both decrease the rate of adsorption as well as the total amount of adsorbed phage in the supernatant (Figure 2G). In addition, attempts to isolate ORF6 KO ΦNP mutants through chemical mutagenesis proved not possible, suggesting it as an essential protein within the ΦNP infection pathway. Taken together, these results are consistent with the view that ORF6 encodes the putative RBP of ΦNP.

### Identification of a novel enteric RBP and RBP assembly family

**Figure 3:**
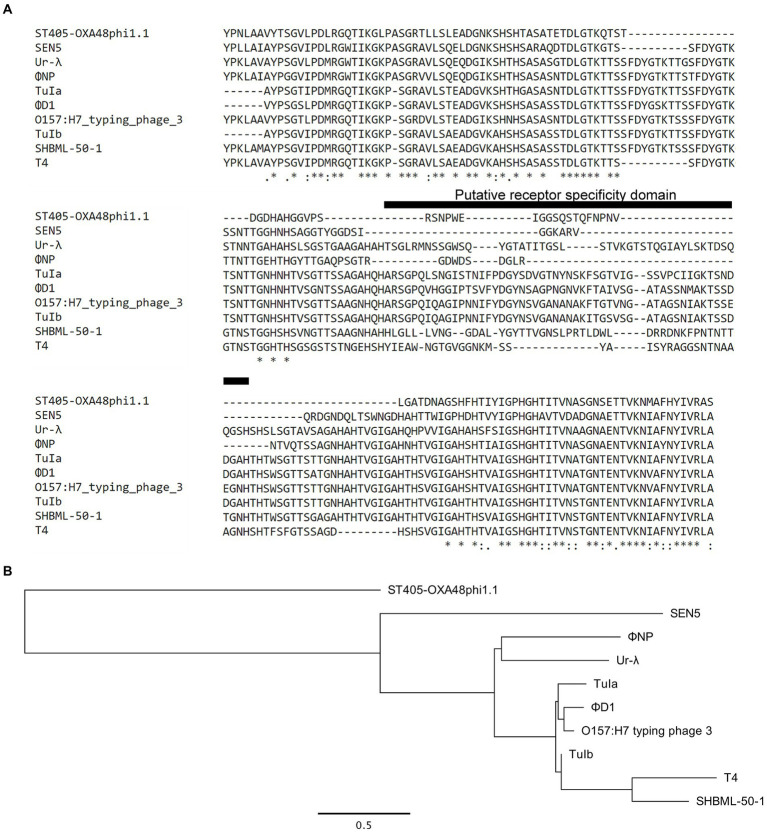
Expansion of bioinformatic search reveals the RBP of ΦNP to be a member of a larger novel enteric RBP family based in a T4- and λ-lineage. **(A)** Amino acid (AA) alignment by ClustalOmega depicting high similarity between ΦNP ORF6 and the distal 215 AAs of the known T4 RBP (gp37), ORF314 of phage λ, and the distal 215 AAs of uncharacterized structural proteins for the *Klebsiella pneumoniae* phage ST405-OXA-48phi1.1, the *E. coli* O157:H7 typing phage 3, the *Yersinia pestis* phage ΦD1, the *Salmonella* spp. phage SEN5, and the *Shigella spp.* phage SHBML-50-1. Note the sequence similarity with primary divergence centered around the putative receptor specificity domain of phage T4. **(B)** Maximum likelihood tree of the putative RBPs of the newly identified enteric RBP family. Trees were constructed using PhyML with default parameters and the General time reversible (GTR) + Gamma model. Branch support was tested using approximate likelihood ratio test (aLRT) based on the Shimodaira-Hasegawa-like (SH-like) procedures and re-tested using 1,000 bootstrap replicates. Tree structures and root positions were verified by Bayesian phylogenetic analysis using BEAST2 under a GTR substitution model, which yielded results consistent with PhyML. Trees are drawn to scale. Scale bars represent the number of substitutions per site.

Further bioinformatic interrogation revealed high conservation between a ~ 215 AA region of the tail fiber encoding ORF314 of phage Ur-λ and ORF6. In addition, protein alignments suggested that ΦNP, as well as the putative tail proteins of over 200 other phage including the *Klebsiella pneumoniae* phage ST405-OXA-48phi1.1, the *E. coli* O157:H7 typing phage 3, the *Yersinia pestis* phage ΦD1, the *Salmonella* spp. phage SEN5, and the *Shigella spp.* phage SHBML-50-1 are members of this same lineage (Figures 3A,B). A full table of hits can be found in Supplementary Table 1 (note: there are 505 homology hits for over 200 unique bacteriophage due to repeat hits). Notably, protein level homology was only seen within the last ~ 215 AAs of each phage tail fiber. Moreover, sequence divergence was seen in the same region of the T4 LTF shown to be responsible for its OmpC/LPS receptor specificity (Montag et al., 1990).

**Figure 4:**
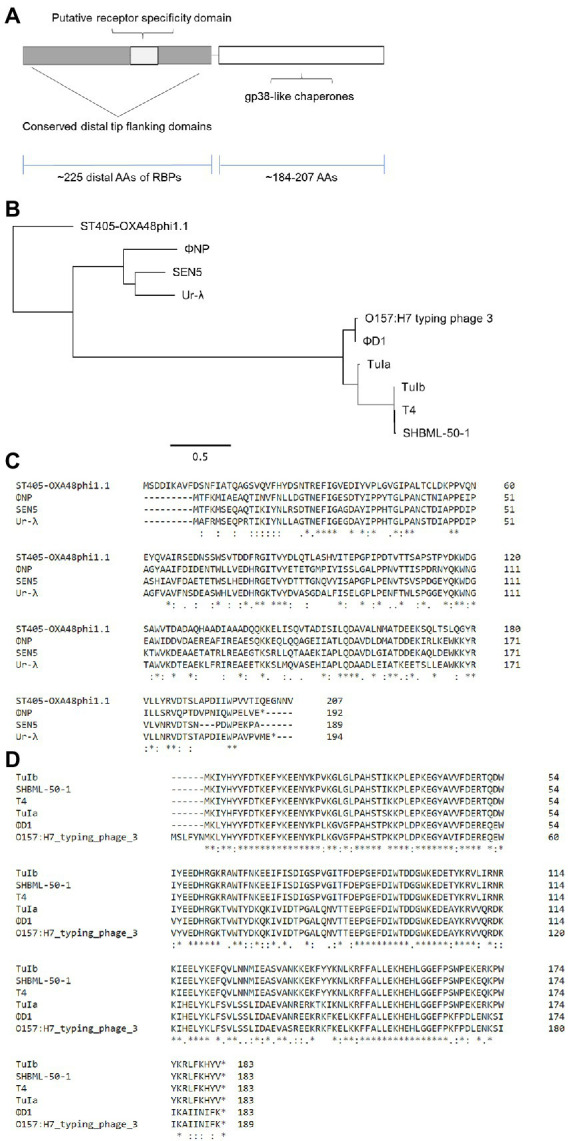
Phylogenetic analysis reveals conservation of both RBP and RBP assembly proteins within the newly identified enteric RBP family. **(A,B)** Amino acid (AA) alignment by ClustalOmega depicting high similarity between downstream structural ORF5 of ΦNP, the known tail fiber assembly chaperone protein of T4 (gp38; downstream of gp37), and uncharacterized structural proteins adject to the putative RBPs of TuIa, TuIb, *Klebsiella pneumoniae* phage ST405-OXA-48phi1.1, the *E. coli* O157:H7 typing phage 3, *Yersinia pestis* phage ΦD1, the *Salmonella spp.* phage SEN5, and the *Shigella spp.* phage SHBML-50-1. When aligned, two clear families emerged: one containing ΦNP, SEN5, Ur-λ, and ST405-OXA-48phi1.1 **(A)** and a more recently evolved family containing T4, TuIa, TuIb, the O157:H7 typing phage 3, ΦD1, and SHBML-50-1 **(B)**. **(C)** Maximum likelihood tree of the upstream structural assembly proteins demonstrating the clear divergence of the two RBP assembly families. Trees were constructed as previously described (Figure 3B). **(D)** A schematic showing the organization of the RBP and gp38-like tail fiber assembly family. Notably, putative tail fiber assembly protein sizes were relatively conserved, ranging from 184 to 207 AAs. In addition, tail fiber assembly coding regions were always found directly downstream of the putative RBP family members.

Furthermore, when we extended protein-level comparison to the immediate upstream and downstream structural modules of ΦNP, ORF5 of ΦNP, predicted to encode a structural assembly protein, showed striking homology to the T4 protein gp38. Indeed, this conservation was present within each of the other phages of the RBP lineage which we examined prior (Figures 4A–D). Earlier studies have shown gp38 to be an essential tail fiber assembly chaperone responsible for mediating the correct trimeric folding of gp37 (Beckendorf et al., 1973; Hashemolhosseini et al., 1996; Leiman et al., 2010). As such, it seems likely that versatility and modularity of this family of RBP tips is, at least in part, due to a conserved assembly protein found directly upstream of the RBP coding region (Figure 4D). When phylogenetically examined, two clear families of assembly proteins emerged: (1) one containing ΦNP, SEN5, Ur-λ, and ST405-OXA-48phi1.1 (Figures 4A,C) and (2) a more recently evolved family containing T4, TuIa, TuIb, the O157:H7 typing phage 3, ΦD1, and SHBML-50-1 (Figures 4B,C). Excepting Ur-λ, with whole genome alignment suggesting large modules of ΦNP morphogenesis (ORFs 5–7, 21 and, 23–26) are of Ur-λ descent (Magaziner et al., 2019), low translational similarity exists between the remainder of the whole phage genomes of ΦNP to other RBP family members (Supplementary Figure 1). We examined the other structural ORFs of several dozen of the phages within this RBP family and found no further homology at any level, including auxiliary tail fiber domains. This would suggest that this RBP and tail fiber assembly protein lineage is not constrained functionally by phage morphology or unique structural proteins motifs but represents an advantageous and conserved absorption module in phage evolution.

## Discussion

The advent of widespread multidrug antibacterial resistance in emerging pathogens has led to a renewed interest in therapeutic alternatives to small molecule drugs. One such revitalized area of focus has been in bacteriophage, or phage, therapy. The primary limitations of phage therapy stem from the incredible host range specificity displayed by phages; in turn this leads to large “cocktails” of therapeutic phages and lengthy screening periods required to treat a small range of pathogenic strains. With impracticalities behind rigorous characterization of each naturally occurring phage used in each “cocktail,” there can often be unforeseen downstream consequences on host physiology and microbiota as well as risks of adverse pathogen evolution in the event of temperate bacteriophage inclusion within the cocktail. To combat this, recent approaches have focused on generating single, well-characterized strictly lytic phages capable of targeting multiple, specific bacterial hosts through exploitation of RBP modularity. Here we identified a new RBP family containing over 200 unique sequenced bacteriophage members including the here elucidated RBP of *Citrobacter rodentium* temperate phage ΦNP as well as the RBPs of phages T4, Ur-λ, the *Klebsiella pneumoniae* phage ST405-OXA-48phi1.1, the *E. coli* O157:H7 typing phage 3, the *Yersinia pestis* phage ΦD1, the *Salmonella* spp. phage SEN5, and the *Shigella* spp. phage SHBML-50-1.

Previously, we had extensively characterized the ten prophages of non-motile, Gram-negative, enteric bacterium *Citrobacter rodentium*, including the temperate phage ΦNP (Magaziner et al., 2019). Here we used these prior insights to identify the uncharacterized structural protein ORF6 of ΦNP as having extensive AA similarity to the known RBP of phage T4, gp37. This was initially quite exciting as T4 represents one of the most, if not the most, well characterized lytic bacteriophages of the last 100 years (Karam and Miller, 2010). Identifying a potential family of RBPs which might serve to expand T4’s host range would be incredibly valuable in the effort to design a modulated therapeutic alternative to naturally occurring “phage-cocktails.” Through adsorption kinetics assays and use of a sfGFP-tagged version of this structural protein and predicted tail fiber we identified ORF6 to be the putative RBP of ΦNP. Corroborating the essential role of ORF6 in ΦNP infection, attempts *via* chemical mutagenesis to obtain an ORF6 KO were not successful. Notably, this approach of fluorescence localization and adsorption occlusion of WT phage virions in the presence of the tagged tail fiber represents a facile means of examining supposed RBPs of other phages.

We also identified that the putative RBP of ΦNP was closely relate to ORF314 of phage Ur-λ. However, it is well known that the RBP of the λ bacteriophage is the tail fiber encoded by gene J which allows binding to its putative receptor LamB (Werts et al., 1994). The early laboratory history of phage λ explains this finding. The “true” wild type prophage λ is designated as Ur-λ; yet, the most common laboratory strain referred to as WT-λ is in fact a variant named PaPa-λ (Pasadena/Paris; Casjens and Hendrix, 2015). PaPa-λ, while possessing the tail fiber encoded by gene J, lacks the short tail fibers (*stf* gene) of Ur-λ; this *stf* gene deletion was first identified by Sanger and colleagues to be the result of a frameshift, generating two truncated reading frames in PaPa-λ ORF401 and ORF314 (Karam and Miller, 2010). Rather interestingly, *stf* has been shown to encode tail fibers that specifically absorb to LPS residues, not unlike gp37 of phage T4 and ORF6 ΦNP (Sanger et al., 1982; Hendrix and Duda, 1992). It is thus likely that a unifying feature of this RBP family may be LPS specificity (either as secondary to a putative receptor, as with T4 and Ur-λ, or as the sole LPS residue-specific binding site, as with ΦNP). This universality of LPS binding might help explain why this novel RBP family is conserved across such varying bacteriophage.

Further protein level alignments suggested a RBP lineage including structural protein conservation in the sequenced genomes of over 200 other bacteriophages, predominantly of those whose bacterial hosts are of the *Enterobacteriaceae*. Notably, protein level homology was only seen within the last ~ 215 AAs of each phage structural protein and likely tail fiber, with primary sequence divergence centered around the putative receptor specificity domain of T4 gp37 product LTF (shown to be responsible for T4’s OmpC/LPS receptor specificity; Montag et al., 1990). We then extended protein-level comparison to the immediate upstream and downstream structural modules of these phages to the T4 protein gp38, a key chaperone for tail fiber folding. Similar to the tail fiber itself, ΦNP ORF5- and T4 gp38-like chaperones encoded genomically adjacent to the tail fibers were identified in each RBP family member. This included the well characterized *stf* (tail fiber) and *lyfa* (chaperone) of Ur-λ, suggesting that the RBP family included both the tail fiber and tail fiber assembly module. Fascinatingly, an early study showed that the N-terminal region of Ur-λ *stf* can functionally substitute for gp37 in receptor binding; similarly the Ur-λ tail fiber assembly protein (*ltfa*) can functionally substitute for gp38 in mediating the correct folding of gp37 (Montag et al., 1989). Notably, no other homology with any other tail fiber or structural proteins was observed between RBP family phage at either the nucleotide or amino acid level. In addition, phage genome sizes (and thus likely, corresponding structural sizes and variations) ranged widely from as small as 20 kb base pairs to as large as several 100 kb base pairs. While modularity is a core feature of bacteriophage evolution and expansion (Lima-Mendez et al., 2011), this was still surprising given the incredibly dissonant whole genome profiles of each member. With the majority family predominantly constrained to enterics, the wide prevalence of this RBP family speaks to its versatility and efficacy within the host and gut environment.

Testament to the overarching limitations present with traditional phage therapy, only 5 (ΦNP, T4, TuIa, TuIb, and Ur-λ) of the over 200 members of the RBP family here reported are well characterized with known extracellular bacterial receptors. Interestingly, despite RBP and chaperone conservation, ΦNP, T4, TuIa, TuIb, and Ur-λ rely on different extracellular receptors for adsorption, LPS residue GlcII, OmpC, OmpF, OmpC/LamB, and LamB, respectively, with all excepting ΦNP possessing a secondary, non-essential, irreversible binding affinity for LPS elements (Henning and Jann, 1979; Hendrix and Duda, 1992; Magaziner et al., 2019). These differing receptor specificities are likely explained by the ~ 30–70 AA unique, receptor-specific domain of each RBS sandwiched by the conserved RBP-tail fiber tip domain outlined in this study.

Taken together with previous studies, our findings highlight a conserved modular domain for bacteriophage host range specificity. It is worth noting, however, that while initial absorption to a bacterial surface often serves as the greatest filter to host range specificity there are a suite of other considerations in the engineering of successful recombinant phage. For example, proper DNA injection and transfer, native bacterial anti-viral defenses (e.g., CRISPR-Cas9 or Toxin-Antitoxin altruistic suicide cassettes) or proper development of phage can all be rate-limiting steps in bacteriophage infection and propagation post-absorption (Pires et al., 2016; Łobocka et al., 2021).Various phage engineering techniques have been pioneered to address these issues including a wide range of phage genome engineering strategies to circumvent native antibacterial resistances or non-compatible genomic islands or GC contents for more efficient phage co-opting of bacterial host replication machinery (Duong et al., 2020; Mahler et al., 2022). This having been said, recombinant expression of the modular RBP and tail fiber assembly lineage reported here might serve to help expand the host range of several very well characterized phages to several clinically important human pathogens for use in therapeutic applications and be of notable interest for phage-based technologies at large.

## Data availability statement

The raw data supporting the conclusions of this article will be made available by the authors, without undue reservation.

## Author contributions

SM and GS: conceptualization, methodology, project administration, visualization, writing: original draft preparation, and writing: review and editing. SM: data curation, formal analysis, investigation, and validation. GS: funding acquisition and supervision. All authors contributed to the article and approved the submitted version.

## Funding

This work was supported by the Biotechnology and Biological Sciences Research Council (BBSRC, UKRI) through awards BB/W000105/1, BB/T006668/1, and PHJZ/744 to GS.

## Conflict of interest

The authors declare that the research was conducted in the absence of any commercial or financial relationships that could be construed as a potential conflict of interest.

## Publisher’s note

All claims expressed in this article are solely those of the authors and do not necessarily represent those of their affiliated organizations, or those of the publisher, the editors and the reviewers. Any product that may be evaluated in this article, or claim that may be made by its manufacturer, is not guaranteed or endorsed by the publisher.

## Footnotes

^1^ https://blast.ncbi.nlm.nih.gov/Blast.cgi
