# Supplementary material for: A novel T4- and λ-based receptor binding protein family for bacteriophage therapy host range engineering
Source: Front Microbiol. 2022 Oct 31;13:1010330. doi: 10.3389/fmicb.2022.1010330 (PMC9659904; doi:10.3389/fmicb.2022.1010330)
Supplement: Supplementary file 1 [file Data_Sheet_1.DOCX]

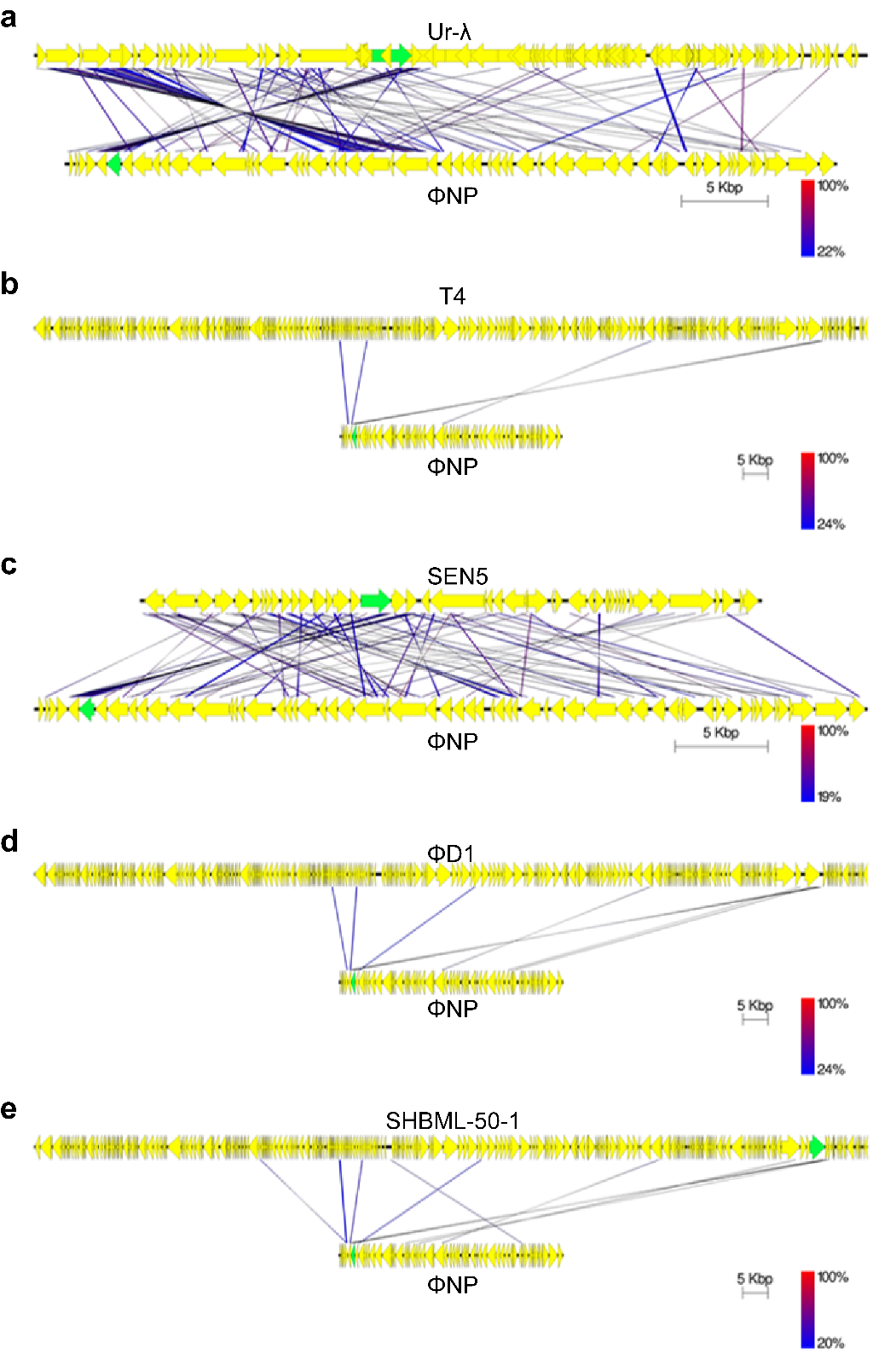


**Supplementary Figure 1. Whole genome analysis of members of the novel RBP shows little global sequence conservation among phage. a-e)** Non-exhaustive whole genome alignment by blastn of ΦNP to family members Ur-λ (a), T4 (b), SEN5 (c), ΦD1 (d),and SHBML-50-1 (e). Excepting Ur- λ, with whole genome alignment suggesting large modules of ΦNP morphogenesis (ORFs 5-7, 21 and, 23-26) are of Ur-λ descent, low translational similarity exists between the remainder of the whole phage genomes of ΦNP to other RBP family members. DNA is represented by black bars with coding regions as yellow arrows. RBP and tail fiber assembly coding regions are green. Similarity was determined by blastn with high (red) and low (blue) sequence match annotated by color. Scale bars are 5 kbp.
